# CAR-NK Cells in the Treatment of Solid Tumors

**DOI:** 10.3390/ijms22115899

**Published:** 2021-05-31

**Authors:** Ewa Wrona, Maciej Borowiec, Piotr Potemski

**Affiliations:** 1 Department of Clinical and Laboratory Genetics, Medical University of Lodz, 92-231 Lodz, Poland; maciej.borowiec@umed.lodz.pl; 2 Department of Chemotherapy, Medical University of Lodz, Copernicus Memorial Hospital, 93-513 Lodz, Poland; piotr.potemski@umed.lodz.pl

**Keywords:** chimeric antigen receptor (CAR), natural killer (NK) cell, solid tumors, review

## Abstract

CAR-T (chimeric antigen receptor T) cells have emerged as a milestone in the treatment of patients with refractory B-cell neoplasms. However, despite having unprecedented efficacy against hematological malignancies, the treatment is far from flawless. Its greatest drawbacks arise from a challenging and expensive production process, strict patient eligibility criteria and serious toxicity profile. One possible solution, supported by robust research, is the replacement of T lymphocytes with NK cells for CAR expression. NK cells seem to be an attractive vehicle for CAR expression as they can be derived from multiple sources and safely infused regardless of donor–patient matching, which greatly reduces the cost of the treatment. CAR-NK cells are known to be effective against hematological malignancies, and a growing number of preclinical findings indicate that they have activity against non-hematological neoplasms. Here, we present a thorough overview of the current state of knowledge regarding the use of CAR-NK cells in treating various solid tumors.

## 1. Introduction

The immune system plays a major role in the growth and progression of malignancies. Under the influence of the immunosuppressive tumor microenvironment (TME), populations of immune competent cells are reprogrammed to support the aggressiveness and the metastatic ability of the tumor, and its resistance to treatment. These rarely preserve their natural anti-tumor activity. Many studies have examined the alterations of the immune system for an improvement of its tumor-killing abilities, resulting in the development of a range of adoptive cell therapies (ACTs). Research on tumor-infiltrating lymphocytes (TILs), T cells expressing genetically modified receptors (TCRs) and chimeric antigen receptors (CARs), in particular, has drastically changed the outcome for many cancer patients [1].

The transduction of CARs into patient-derived T cells became a milestone for treating patients with hematological neoplasms in recent years. CAR-T cell therapy became one of the most sophisticated examples of personalized medicine. However, despite its confirmed anti-tumor activity, CAR-T cell therapy has several drawbacks: production is time-consuming and requires advanced technical support, and the therapy is very expensive and has a significant toxicity profile [2]. In the light of these shortcomings, the substitution of T cells with NK cells, i.e., CAR-NK therapy, may be a better alternative, one that maintains the effectivity of CAR-T but is free of its drawbacks. The facts that CAR-NK cell therapy does not require HLA or KIR (killer Ig-like receptor) compatibility, does not entail severe toxicities [3] and can be derived on a mass scale from several sources make it a truly off-the-shelf product and a very promising natural successor to CAR-T cell therapy [4].

Although CAR-NK cells might replace CAR-Ts in hematological cancer treatment, limited data exist on its potential in solid tumor treatment. Despite large similarities between CAR-NK and CAR-T cell therapies, the inefficiency of CAR-T cells in preliminary solid tumors studies should not be extrapolated on CAR-NK therapy. CAR-NK cells have already been widely tested in glioblastoma (GBM), breast, ovarian and pancreatic cancer, among others, and it appears that the natural properties of NK cells provide CAR-NK cells with a high chance of succeeding in solid cancer therapy.

In response to the mounting data on this emerging therapy, the present paper provides a thorough review of the technical issues, molecular background and current preclinical and clinical data associated with CAR-NK cell applications in cancer treatment.

## 2. Production Process of CAR-NK Cells

The production protocols of CAR-NK cells largely overlap with those developed for CAR-T cells. NK cells need to incorporate a DNA strand with a prepared template for the CAR construct. As addressed below, major dissimilarities, when compared with CAR-Ts, consider alternative sources of vehicle cells and the need for their further expansion and activation.

### 2.1. Sources of NK Cells for CAR-NK Cell Therapy

NK cells can be obtained for CAR-NK production from a number of sources, e.g., immortalized human NK cell lines, donor peripheral blood, archived samples of umbilical cord blood or supervised differentiation of pluripotent cells. Depending on the origin, harvested NK cells vary with their maturation stage and viability, which is reflected in the diverse anti-tumor effectiveness of the produced CAR-NK cells. However, constant optimization of protocols for deriving NK cells from various sources and scaling up production will inevitably offer a Good Manufacturing Practice (GMP)-compliant off-the-shelf cancer treatment.

Initially, preclinical studies on the CAR-NK cell concept were based on the NK92 cell line, derived from a 50-year-old male with extranodal NK cell lymphoma (ENKL). Since it is relatively easy to expand in vitro, the NK-92 cell line is potentially a source of limitless number of CAR-NK cells. However, as the engineered NK-92 cells are of malignant origin, the cells must be irradiated to limit their in vivo engraftment potential [5]. Although irradiation with 10 Gy limits their ability to proliferate, it also shortens the survival of CAR-NK92 cells in the peripheral blood of the recipient. Another drawback is that they are naturally deprived of the CD16 domain, and are hence unable to trigger ADCC (antibody-dependent cell cytotoxicity), one of the potent intrinsic NK cell anti-tumor mechanisms [6]. In addition, a recent study suggests that the host peripheral blood mononuclear cells may demonstrate a possible cytotoxic reaction against CAR-transduced NK-92 cells, which could explain their recently reported inferior efficacy compared to CAR-T cell therapy [7]. Apart from NK92, other human cell lines such as NKL, KHYG-1, YTS or NKG have also been evaluated as possible alternatives [8].

Another source of NK cells is the peripheral blood (PB) of donors, where mature NK cells can be easily harvested with no need for HLA/KIR matching; however, relatively few cells can be obtained from each donation (NK cell portion is oscillating around 10% of peripheral blood mononuclear cells (PBMCs)) [9]. PB is rich in mature NK cells that unfortunately do not expand easily in vitro. These are characterized by CD56^bright^/CD16^+^ expression and constitute approximately 90% of the NK cell population in peripheral blood in various stages of maturation, from maturing NKG2A^+^KIR^-^ and NKG2A^+^KIR^+^ to fully mature NKG2A^-^KIR^+^ NK cells, which makes PB-derived CAR-NK cells heterogeneous and difficult to standardize [10,11,12]. On the other hand, the cells obtained from PB respond more effectively and persist in circulation for longer than NKs from other sources [13].

NK cells can also be derived from umbilical cord blood (UCB). Large counts of NK cells can be obtained from numerous archived samples of UCB. NK cells constitute about 30% of the lymphocytes in UCB [14,15]; however, in contrast to PB, UCB-derived NK cells generally present an immature phenotype (CD56^dim^/CD16^−^) [16], and hence, inferior cytotoxic capabilities. Despite this, they also demonstrate greater potential to expand than PB-derived NK cells [13].

Finally, the stimulation of immunocompetent progenitor stem cells (iPSC) could also be a promising source of NK cells. In this case, iPSCs are harvested from the mobilized peripheral blood of the donor or from UCB. The CAR construct is transduced into iPSCs, which are then differentiated into CAR-expressing NK cells by incubation in a cytokine cocktail of SCF, VEGF, BMP4, IL3, IL-15, IL-7 and FLT3L [17]. The major virtue of iPSC-derived CAR-NK cells is the potential to produce large numbers of homogeneous CAR-NK cells from one iPSC. On the other hand, this technology generates cells with an immature, less cytotoxic phenotype, similar to UCB-derived NK cells [18,19].

No available source of NK cells is deprived of defects. However, the phenotypes of harvested NK cells can be additionally modified to improve their cytotoxicity and survival in vivo.

### 2.2. Maximizing CAR-NK Cell Survival

Apart from choosing the optimal source of NK cells, an essential goal in CAR-NK production is to maximize their cytotoxic capabilities and extend their persistence in the peripheral blood. Unmodified NK cells were detected in the circulation for only up to one week after infusion [20,21], and NK92 cells for up to 48 h post infusion, which seems to be insufficient for CAR-NK therapy. Although the short survival of NK cells limits the efficacy of the therapy, it also reduces their potential to trigger severely adverse events.

Historically, IL-2 or IL-15 were administered after NK cell infusion to stimulate their expansion and activation in vivo; however, this procedure entailed a high risk of capillary leak syndrome and shortly led to the exhaustion of over-stimulated NK cells (cytotoxicity and cytokine production impairment, and reduced proliferative capabilities) [22,23]. High NK cell counts can also be maintained in vivo by introducing a transgene of an interleukin, which results in constant self-activation of NK cells with no need for exogenous injections. Introduction of membrane-bound IL-15 (mbIL-15) into CAR-NK construct was shown to increase NK cell proliferation and survival, although it failed to be curative [24]. Finally, the therapeutic potential of iC9/CAR19/mbIL-15 construct in UCB-derived NK cells has been largely improved by knocking out the *CISH* gene in transduced NK cells when tested against CD19-Raji lymphoma cells. *CISH* encodes CIS (cytokine-inducible SH2-containing protein), which is responsible for suppressing cytokine signaling upon IL-2 and IL-15 stimulation. As a key cytokine-related immune checkpoint inhibitor, CIS decreases CAR-NK cells cytotoxicity and anti-tumor activity [25].

The issue of in vivo persistence is also addressed by stimulating a memory-like CAR-NK cell phenotype. Historically, memory-like NK cells were discovered as a consequence of CMV (cytomegalovirus) infection. CMV was found to induce expansion of an NK cell subpopulation with CD94/NKG2C overexpression and decreased expression of PD-1, TIGIT and NKG2A checkpoint receptors [26,27]. The CD56^dim^/CD16^bright^/CD94/NKG2C cells presented increased proliferation, degranulation and high production of IFN-γ and TNFα upon activation [28]. Pre-activation of NK cells by incubation in multi-cytokine medium (16h culture with IL-12, IL-15 and IL-18) induces the formation of memory-like NK cells with similar abilities and characteristics as CMV-induced cells [29]. Such NK cells present durable and enhanced response, as well as a gene expression profile and methylation pattern similar to memory CD8^+^ T cells [26,28,29,30].

Preclinical data suggest that inserting CAR into a memory-like NK cell could increase its survival and cytotoxicity [31]. The obtained memory-like CAR-NK cells demonstrate superior degranulation and IFN-γ-based response against acute myeloid leukemia (AML) cell lines and in vivo mice models. Memory-like NK cells are reported to demonstrate better cancer cell killing and to mediate stronger ADCC; they can also survive longer and resist suppression by the MDSC and Tregs that are abundant in the solid tumor TME [32,33]. A preclinical in vivo study of CD19-directed memory-like CAR-NK cells (anti-CD19.CD8a.4-1BB.CD3ζ.CAR-PBNK) found the therapy to be safe and demonstrated increased efficacy against lymphoma compared with conventional CD19-directed CAR-NK cells. Interestingly, despite the low efficiency of CAR structure transduction (15–25%) into memory-like NK cells, they have been found to greatly expand in vivo in response to the target CD19 antigen, reaching up to 40–90% of the circulating NK cell population [31].

### 2.3. Expansion and Activation

The major hurdle in CAR-NK cell production, regardless of the source of NK cells, is that they all require expansion and activation prior to infusion. Multiple protocols have been developed to obtain a clinically useful count of activated NK cells. Nevertheless, producing a suspension of homogeneous, GMP-compliant NK cells demonstrating a similar maturity stage and phenotype remains a great challenge. Avoiding contamination from T lymphocytes, which are more responsive to the available expansion protocols and could ignite GvHD after unmatched infusion, also presents a challenge. This can be achieved either by incubating NK cells in a cocktail of interleukins and/or through culture with artificial antigen-presenting cells (aAPC) called feeder cells.

In initial expansion and activation protocols, NK cells were incubated with IL-2, both in in vitro culture and as in vivo infusions [34,35,36]. Later, this was replaced with IL-2-diphtheria toxin fusion protein; this was found to be a superior alternative, since it caused intensive, selective expansion of NK cells with no stimulation of Treg lymphocytes that express the IL-2Rα receptor and are co-expanded by incubation with unmodified IL-2 [37,38]. Similarly, IL-15 was also used to expand NK cells in vitro [39] and in vivo [40]. This procedure resulted in large numbers of fully functional NK cells; however, it led shortly to its exhaustion [41]. Nevertheless, protocols based only on incubation with interleukins were found to be less effective than culturing with an aAPC.

Historically, NK cell incubation with irradiated B-lymphoblastoid cells resulted in 25-fold expansion over a two-week period [42], while 30-fold expansion was reported after 18 days of culture with monocytes and IL-2 [43]. More efficient protocols are currently in development; however, it is worth remembering that implementing the feeder cell-based protocol leads to a risk of contamination and requires separation of expanded NK cells from feeder cells prior to infusion. The most commonly used approach for the expansion and activation of CAR-NK cells is incubation with feeder cells, e.g., the Wilms tumor cell line, irradiated K562 cells or the human B-lymphoblastoid cell line 721.221.

NK cell culture with HLA class I-negative Wilms tumor cell line resulted in 400-fold expansion after a two-week period [43]. Superior results were achieved with an unmodified K562 cell line culture that was shown to expand NK cells and increase their cytotoxic activity [44]. Incubation of PB-derived NK cells with modified K562 cells expressing 4-1BBL and mbIL-15 (K562-mbIL-15.4-1BBL cells) resulted in 1000-fold expansion over a three-week period with no co-stimulation of T lymphocytes [45]. Similar results were reported using K562-mbIL-21.4-1BBL cells [46]. Yet another approach is to genetically modify HLA-A- and HLA-B-negative K562 cells to express CD48, 4-1BBL and mbIL-21. Incubation of unmodified NK cells and CAR-NK UCB-derived cells with the described K562-CD48.4-1BBL.mbIL-21 cells resulted in >900-fold expansion over two weeks. This resulted in the creation of a homogeneous NK cell population of high purity (99.9%) with no signs of T cell expansion. Moreover, both unmodified and CAR-expressing NK cells obtained did not show any signs of exhaustion [47].

Most recently, human B-lymphoblastoid cell line 721.221 expressing mbIL-21 (721.221-mIL-21) was shown to expand NK cells more efficiently than K562-mIL-21 feeder cells. The procedure enabled the production of a homogeneous population of non-exhausted NK cells with a memory-like phenotype [9,44]. The presented results are highly promising and suggest that this could be the most optimal method for CAR-NK cell production.

## 3. The Therapeutic Mechanisms of CAR-NK Cells

The replacement of CAR-T lymphocytes with CAR-NK cells seems to result in significantly greater therapeutic effectiveness. Apart from the anti-tumor activity entailed by CAR expression, NK cells have a natural ability to kill cancer cells. Hence, CAR-NK cytotoxicity can be modulated by optimizing not only the CAR design but also the intrinsic activity of NK cells.

### 3.1. CAR-Independent Response

The anti-tumor activity of intrinsic NK cells is versatile (Table 1.). In contrast to T lymphocytes, activated NK cells are not dependent on antigen recognition for cytotoxicity initiation. Their tumor killing capabilities act by stimulating cell lysis through the degranulation and release of perforin and granzyme. NK cells also express transmembrane receptors, e.g., the natural cytotoxicity receptors (NCRs), KIRs, NKG2D or DNAM-1, which enforce caspase-mediated apoptosis of recognized cancer cells. IFN-γ secretion results in the recruitment of macrophages and dendritic cells contributing to alternative anti-tumor mechanisms. Additionally, NK cells are able to provoke cancer cell killing mediated by ADCC [48,49,50]. Mature NK cells express CD16 receptor that is a key factor in ADCC. It recognizes the Fc fragments of an immunoglobulin G (IgG) bound to an epitope on the surface of the tumor cell. This mechanism has been found to be involved in the success of anti-HER2 and anti-EGFR therapy in solid tumors [51]. NK cell infiltration in the TME is associated with better outcome in multiple solid cancers, which is primarily explained by their ability to trigger ADCC [51,52]. Apart from supplementing NK cells with optimal CAR structures, several studies have also attempted to improve their intrinsic killing capabilities.

NKG2D is an activating receptor responsible for provoking caspase-mediated apoptosis [53]. In in vitro and in vivo models of human pancreatic and gastric cancer, NKG2D was found to determine the recognition of cancer stem cells (CSCs) by NK cells, depending on the expression of CD133 and CD24 [54,55]. NKG2D-dependent cytotoxicity was especially intensified against tumors infiltrated by NK cells with up-regulated NKG2D expression, as reflected by a superior outcome in these patients. NKG2D can also induce cytotoxicity by NK cells by recognizing *stressed cell ligands* on tumor cells, e.g., MICA and MICB. Their expression on the cancer cell surface stems from hypoxia, nutrition deprivation, chemotherapy or irradiation [56].

A study on patient-derived sarcoma tissue indicated that despite the high expression of CD112 and/or CD155 (DNAM-1 ligands), the number of tumor-infiltrating NK cells was low, especially when compared with the surrounding healthy tissue. Altering NK92 cells to overexpress DNAM-1 led to increased trafficking, efficient degranulation and potent anti-sarcoma activity in all tested patient-derived sarcoma cell samples. DNAM-1-overexpressing NK92 cells also demonstrated enhanced degranulation against several solid tumor cell lines, including prostate cancer, pancreatic cancer, and colon and lung cancer cell lines [57].

Additionally, reduction of KIR-mediated self-inhibition escalates the cytotoxicity of NK cells and enhances ADCC. NK cells with downregulation of KIR2DL2/L3, KIR2DL1 and KIR3DL1 presented less sensitivity to inhibitory signals from HLA-I presenting cancer cell lines, resulting in superior anti-tumor activity [58].

### 3.2. CAR-Dependent Response and CAR Constructs for NK Cells

Initial CAR-NK cell studies were based on the direct transfer of CAR structures tailored to T cell-based therapies. These were built of domains relevant for T lymphocyte signaling pathways, being mainly CD3ζ as a signaling domain and 4-1BB or CD28 as co-stimulatory domains (Table 2.) [59]. Several research groups suggest that re-designing CARs with a set of domains relevant to NK cell signaling could increase its anti-tumor efficacy. In fact, CARs implementing NK-specific 2B4, DAP-10 or DAP-12 particles as co-stimulatory domains are characterized by improved cytotoxicity and higher IFN-γ secretion than CAR-T-tailored constructs (Table 2).

A direct comparison of several CAR structures revealed NK cell-tailored CARs result in superior anti-tumor effectivity than those transferred directly from CAR-T cell studies. A study found that CAR-NK cells containing NK-specific, DNAM1 and/or 2B4 co-stimulatory receptors demonstrate higher cytotoxicity against several hepatocellular cancer cell lines (HEpG2, Hep3B, L-02, Huh7) than CAR-T cell-derived constructs. The tested CAR structures were CD3ζ alone, CD28.CD3ζ (CAR-T cell derived), DNAM1.CD3ζ, 2B4.CD3ζ and DNAM1.2B4.CD3ζ (DNAM1 and 2B4 are both NK-cell-specific co-stimulatory receptors), which had been transferred into NK92 cells and directed against the GPC3 antigen [60]. In addition, CAR-T structures were also compared with CAR-NK structures against neoplasms originating from T lymphocytes. When expressed on NK92 cells, the CD5-directed 2B4.CD3ζ.CAR construct performed better at mediating effective cytotoxicity against Jurkat and MOLT-4 cell lines than the T cell-tailored 4-1BB.CD3ζ.CAR structure [61].

Elsewhere, iPSC-derived NK cells were transduced with CAR structures with variable combinations of NK-specific receptors, e.g., CD16, 2B4, NKp44, DAP10, NKG2D and DAP12. Out of all tested designs, those with activating receptor NKG2D and co-stimulatory receptor 2B4 demonstrated the highest cytotoxicity in xenograft ovarian mouse models expressing mesothelin. Moreover, treatment with mesothelin-directed NKG2D.2B4.CD3ζ.CAR-NK cells offered superior survival prolongation and tumor volume reduction compared to the mesothelin-directed CAR-T cells [17].

PSCA-directed CAR-NK with DAP12 incorporated as a co-stimulatory receptor was also found to demonstrate potent anti-tumor activity. The DAP12-based construct showed superior cytotoxicity in xenograft mouse models of prostate cancer than the CD3ζ-based CAR-NK. Interestingly, higher cytotoxicity was noted against the KIR/HLA-mismatched than the KIR/HLA-matched tumor cells [62].

### 3.3. The Advancement of Nanobodies

A considerable improvement in recent years is the incorporation of Camelidae-derived nanobodies (Nb) into CAR constructs. Regular CARs contain scFv fragments with variable heavy-chain V_H_ and light-chain V_L_ murine monoclonal antibodies [59]. However, as the variable fragments lack the stabilization and scaffolding normally provided by constant chains CH_1_ and C_L_, it is possible that they can collapse, fold and, subsequently, fail to recognize targeted cancer antigen [63,64]. Nanobodies, on the other hand, are miniaturized equivalents of murine antibodies composed only of a highly soluble heavy-chain single domain. Its small size (approximately 15 kDa compared to >25 kDa of a murine scFv fragment) and physical properties allow it to be recombined into multimers with greater binding strength and specificity while maintaining effective penetration with its small size and stable domain structure [65]. With these properties, nanobodies are a promising innovation as an Nb-CAR-based therapy.

Two studies analyzed the preclinical application of Nb-CAR-NK cell therapy [66,67]. Human CD38-directed nanobodies incorporated into a CAR particle (anti-CD38Nb.CD28.4-1BB.CD3ζ.CAR-NK92) demonstrated anti-tumor activity. Both CD38-expressing multiple myeloma (MM) cell lines and primary patient-derived MM bone marrow samples were successfully depleted by CD38-directed Nb-CAR-NK92 cell therapy [66].

Another example of the application of nanobodies was given by You et al. analyzing the application of CD7-directed CAR-NK92MI constructs, and their multimers, against T cell-originating malignancies. In a direct comparison, doubled dCD7Nb.CAR-NK92MI therapy showed superior cytotoxicity against T cell lines and xenograft mouse models of primary T cell tumors over the monovalent CD7Nb.CAR structure [67]. These initial preclinical findings are highly promising, although a study comparing anti-tumor Nb-CAR-NK and conventional CAR-NK cell abilities in solid tumors is still awaited.

### 3.4. CAR Constructs Directed against Malignancies Originating from T Cells

An area of great demand for adoptive cell therapies are malignancies originating from T cells. T cell leukemia and lymphoma blasts share transmembrane receptors with healthy T lymphocytes. In this setting, CAR-T cell therapy directed against T cell-specific receptors would result in fratricide during the production process. Additionally, it is extremely difficult to harvest healthy populations of T lymphocytes from patients with T cell neoplasm without any contamination by malignant cells [68]. With this in mind, applying NK cells to T cell-directed CAR therapy seems to be the perfect solution.

The effectiveness of CARs directed against an alternative T cell-specific antigen, CD7, a marker detected also in 30% of AML blasts, was described in the aforementioned study by You et al. The NK-92MI cell line, characterized by IL-2 independence, has been transduced with either monovalent and bivalent nanobodies CD7Nb-directed CAR constructs (CD7Nb/dCD7Nb-directed CD28.4-1BB.CD3ζ.CAR-NK-92MI) and both presented strong anti-tumor activity [67]. CAR-NK cells directed against CD7 antigen are currently evaluated in a phase I/II clinical trial (NCT02742727). In addition, studies of CD5-directed CD28.4-1BB.CAR-NK92 cell therapy found these constructs to have strong cytotoxicity against T cell acute lymphoblastic leukemia, peripheral T cell lymphoma, Sezary cells and mouse models of Jurkat cell lymphoma [61,69].

## 4. NK Cell Modifications for Solid Tumor Treatment

Several difficulties need to be addressed for successful implementation of CAR-NK therapy in solid tumors. In addition to designing the optimal CAR structure, it is also equally important to make genetic modifications of the intrinsic inhibitory and activatory pathways of NK cells. Such alterations could increase activation and extend the persistence of CAR-NK cells and in the peripheral blood of the recipient. Great efforts have been made to reprogram NK cells to maximize their intratumor trafficking, overcome the immunosuppressive TME and prevent cancer antigen escape.

### 4.1. Intrinsic Pathway Modifications

The NKG2D receptor on NK cells was known to induce granule-dependent cytotoxicity and cell-mediated cytotoxicity. However, its activation was insufficient to trigger IFN-γ production [53]. This insufficiency was addressed by combining NKG2D with a signaling partner, DAP10, for downstream activation of PI3K, which enhanced killing initiation [70]. NKG2D.DAP10.CD3ζ.PBNK was also found to increase the anti-tumor activity of modified NK cells against several cell lines, e.g., osteosarcoma, prostate cancer, colon cancer, gastric cancer, squamous cell lung cancer, hepatocellular carcinoma, breast cancer, rhabdomyosarcoma and neuroblastoma. Compared with activated unmodified NK cells, cells expressing the NKG2D chimeric construct demonstrated superior cytotoxicity, as well as increased cytokine secretion and granule release [71]. Enhanced cytotoxicity was observed in all the tested cell lines; however, the cytotoxic effect was significantly stronger against rhabdomyosarcoma, osteosarcoma and prostate cancer cell lines and moderate against upper gastrointestinal cancer cell lines [71]. Combining large numbers of altered, activating receptors NKG2D with CAR-expressing NK cells could potentiate its effectivity in solid tumor treatment.

Downregulation of inhibitory receptors has also shown promising results in preclinical studies. Silencing the inhibitory NKG2A receptor on NK cells was found to block its signaling and improve NK cell cytotoxicity against HLA-E-expressing cancer cell lines by 40% [72]. Another attractive candidate for modification was the TIGIT (T cell Ig and ITIM), which naturally binds to CD155 and CD112 receptors on cancer cells, induces immunotolerance and inhibits the immune response mediated by NK and T cells. Knockout of TIGIT in NK cells resulted in a promising increase of anti-tumor activity in preclinical melanoma mouse models [73]. In colon cancer mouse models, TIGIT decreased NK cell exhaustion following blockage by directed antibodies or knockout. TIGIT blockade also triggers a durable anti-tumor memory response, as demonstrated by tumor growth inhibition in mouse models re-challenged by colon cancer cell reinfusion [73].

### 4.2. Combating Heterogeneity

Cancer heterogeneity is prevalent across all solid tumors and causes major difficulties for CAR-engineered cell therapies. For example, some cancer cell subpopulations such as cancer stem-like cells (CSCs), which are responsible for treatment resistance and constant progression, lack the CAR-directed antigen and hence evade anti-tumor activity. In addition, antigen escape can occur, resulting in an antigen being hidden in the cancer cell upon CAR-based treatment [74]. Both phenomena are thought to increase the chance of CAR-T cell therapy failure. Nevertheless, the incidence of antigen loss is thought to be limited in the case of CAR-NK cell therapy due to their dual, CAR-dependent and CAR-independent, anti-tumor activity. NK cells, thanks to diversified mechanisms, could be a solution in the treatment of heterogeneous neoplasms.

Heterogeneous tumors are known to evade T cell recognition and elimination, which explains the poor results of CAR-T cell therapies in solid tumor treatment [75,76]. An experimental model of NK cells transduced with PD-L1-directed CAR was designed to overcome the selection of T cell escape variant cancer cells. CAR-NK cells targeting PD-L1 were shown to successfully recognize and eliminate heterogeneous tumor cell populations in a head and neck squamous cell carcinoma (HNSCC) model [77]. These are currently evaluated in a phase II clinical trial of irradiated PD-L1-directed CAR-NK cells (NCT04847466) for the treatment of gastroesophageal junction and HNSCC cancer patients.

CSCs are characterized by self-renewal properties and are responsible for tumor progression and chemoresistance. However, NK cells were found to preferentially kill CSCs in GBM, melanoma, pancreatic, colorectal and breast cancer models. The CSCs were demonstrated to express the stress ligands MICA and MICB, which are recognized by the NKG2D receptor on NK cells and trigger its cytotoxicity [78,79,80,81]. At the time of writing, i.e., April 2021, MICA/B-directed CAR-NK cell therapy has been under development by Fate Therapeutics for the treatment of solid tumors and hematological malignancies [82,83]. Additionally, IFN-γ and TNFα produced by NK cells are known to enforce differentiation of CSCs, resulting in the loss of their chemoresistance and self-renewal abilities [84]. Altogether, NK cells provide a great repertoire of mechanisms directed against cancer heterogeneity.

### 4.3. Strategies against the Suppressive TME

The TME suppresses immunocompetent cells in several ways. Most importantly, tumor-associated cells secrete a range of immunosuppressive factors, e.g., TGFβ, adenosine, IL-10, arginase-1, PGE2 and IDO [85]. TGFβ secretion leads to immunosuppression by reduced recruitment of functional NK cells, downregulates the NKG2D and DNAM1 activating receptors and blocks perforin secretion [86]. Studies on tumor-infiltrating cells show that most NK cells found in the TME are immature and demonstrate low cytotoxic abilities [87,88]; however, this was not observed in CB-derived NK cells expressing dominant-negative-TGFβ receptor II (DNR II), which preferentially binds to TGFβ with no signal transmission. The DNR II-expressing NK cells were not inhibited by TGFβ and were found to retain their killing ability and cytotoxicity against CML, T-ALL and GBM cell lines [89].

Another approach to overcome the immunosuppressive impact of the TME was the NK-tailored chimeric co-stimulatory converting receptor (CCCR). In contrast to CARs, its structure is composed of the extracellular portion of PD1 receptor, NKG2D and 4-1BB co-stimulators. The designed CCCR-NK cells were found to inhibit lung cancer growth in xenograft mice models by switching the immunosuppressive, negative PD1 signal to an activating one [90]. The concomitant expression of CCCRs and CARs by NK cells might significantly improve the effectivity of the traditional CAR-NK therapy.

### 4.4. Trafficking

There is a great need to increase the ability of CAR-NK cells to infiltrate tumor tissue. However, only a few studies have detailed the genetic modifications that have successfully led to the intensified trafficking of NK cells. Overexpression of the CXCR4 receptor resulted in increased tumor infiltration compared with NK cells expressing CAR alone [91,92]. NKG2D.CAR-NK cells, with overexpression of the CXCR1 receptor, demonstrated enhanced in vivo cytotoxicity. CXCR1 is an IL-8 receptor that, upon activation, stimulates cell mobility up an IL-8 gradient. Multiple cancers, including pancreatic and ovarian cancer, are known to secrete IL-8. Its overexpression was found to result in a higher number of tumor-infiltrating CAR-NK cells in mice xenograft models of peritoneal ovarian cancer, in turn resulting in a superior anti-tumor effect [93]. In addition, tumor tissue infiltration with NK cells was correlated with the CXCL16 gradient in a pancreatic cancer mice model. NK cells were shown to successfully reduce the volume of CXCL16-overexpressing tumors [94].

## 5. Success in Hematological Malignancies

Primarily, most successful preclinical and clinical studies on CAR-NK cells have been conducted on hematological malignancies. A milestone in this type of treatment was a recently published report from a phase I/II clinical trial detailing the successful application of CD19-directed CAR-NK cells. Objective responses were observed in 8 of 11 heavily pre-treated lymphoma patients, with 7 of them maintaining complete responses with negative minimal residual disease on day 30 post infusion. The study analyzed UCB-derived NK cells after retroviral transduction with CD28.CD3ζ endodomain, IL-15 and iCas9 (inducible caspase 9) safety switch and activation/expansion with K562 feeder cells and IL-2. The authors postulate 49.0% transduction efficiency (range 22.7–66.5%) [3].

The study confirms the safety and tolerability of CD19-directed CAR-NK therapy. No maximal tolerated dose was reached, despite the doses being as high as 1×10^7^ of CD19-directed CAR-NK cells per kg of body weight [3]. Several preclinical and clinical studies have found CAR-NK cell therapy to be significantly safer than CAR-T cell infusions. Activated CAR-NK cells secrete IFN-γ, GM-CSF and TNFα, which are deprived of the potential to trigger severe adverse events [95]. In the case of CAR-T cells, its activation caused an outburst of multiple cytokines, e.g., IL-1a, IL-1Ra, IL-2, IL-2Ra, IL6, TNFα, MCP-1, IL-8, IL-10 and IL-15, which resulted in severe complications such as cytokine release syndrome (CRS) and neurotoxicity [96]. Since the idea of CAR-NK therapy is strongly based on HLA-mismatched NK cell donors, it was anticipated that GvHD may be an adverse event. Nevertheless, multiple preclinical and clinical studies contradict that HLA-mismatched CAR-NK therapy can lead to GvHD if the infusion is not contaminated with the donor’s T cells [97]. However, in a trial by Liu et al., no patient was found to be affected by GvHD symptoms, despite an HLA/KIR mismatch between donors and enrolled patients, nor was there any case of iCas9, safety switch, use [3].

Additionally, CAR-NK cells were shown to persist in patients’ peripheral blood for at least 12 months after infusion. While these numbers were neither dose-dependent nor HLA-mismatch-dependent, early expansion was reported to be associated with treatment response [3]. The study proves the safe toxicity profile of the tested therapy and suggests non-inferior anti-tumor effectivity of CAR-NK cells. It delivers a strong positive incentive for subsequent clinical trials.

## 6. Preclinical Data on CAR-NK Cells in Solid Tumors

An increasing number of in vitro and in vivo studies have examined the activity of CAR-NK cells against solid tumors, with most preclinical data being available for GBM, breast, ovarian and pancreatic cancer. These results led to the launch of the first CAR-NK cell clinical trials for solid tumor treatment.

### 6.1. Glioblastoma

Of all solid tumors, GBMs have acquired the most extensive body of preclinical data regarding CAR-NK cell treatment. Although NK cells found in GBM tissue have been found to be suppressed by the TME, the fact that approximately 89% of GBM are naturally infiltrated by NK cells indicates that trafficking CAR-NK cells into the tumor tissue is plausible. Unmodified, activated autologous NK cells infused intravenously or intratumorally have already been assessed in a study of nine GBM patients, in which four achieved a partial/mixed response to the tested therapy and three achieved stable disease. The obtained outcomes were transient due to the strong immunosuppressive impact of the TME; however, the findings suggest that NK cells have considerable potential in the GBM treatment [98]. Numerous GBM target antigens have already been tested in CAR-T cell studies, e.g., IL-13Rα2, EGFRvIII, HER-2, EPHA2, CSPG4, CD133, and CD70 [99]. Studies on CAR-NK cells target a similar set of antigens, although they attempt to address the issues identified by CAR-T cells research.

Intravenous infusion of EGFRvIII-directed DAP12.CD3ζ.CAR-NK-YTS cells with CXCR4 receptor overexpression was found to inhibit the growth of GBM tumors in xenograft mouse models and prolonged their survival [91,92]. Several clinical trials with CAR-T cell therapy in GBM patients indicate that high anti-tumor activity entails a swift antigen loss among the cancer cell subpopulation, and despite an initial response, it quickly develops resistance to CAR-T therapy [100,101,102]. A possible solution to antigen loss in GBM was tested in studies of EGFRvIII-directed CAR-NK cell therapy, where it was found to be prevented by treatment with CAR-NK transduced with bispecific CAR constructs targeting both mutated and wild-type EGFR. Dual-specific EGFR- and EGFRvIII-directed CD28.CD3ζ.CAR-NK-92 were administered intratumorally in intracranial GBM xenograft mice models. Compared with monospecific EGFRvIII-directed CAR-NK, the bispecific structure demonstrated superior survival prolongation without antigen escape [103]. An independent study confirmed these conclusions. Intracranial injections of bispecific EGFR- and EGFRvIII-directed CD28.CD3ζ.CAR-NK-92/NKL prolonged the survival of GBM xenograft mouse models and were characterized by increased cytotoxicity and IFN-γ secretion [99].

### 6.2. Breast Cancer

CAR-NK cells targeting several different antigens demonstrated efficacy against breast cancer in in vitro and in vivo models. As the first example, tissue factor (TF) was selected as an appropriate antigen target for CAR-NK design, since it is present in 50–85% of patients with triple-negative breast cancer. A TF-directed CD28.4-1BB.CD3ζ.CAR construct was transduced into NK-92MI (IL-2 independent) cells that were deprived naturally of CD16 receptor, resulting in an impaired ADCC. In the study, the NK-92MI cell line was additionally modified to restore CD16 expression. In tumor-derived xenograft models, the designed CAR-NK cells presented superior anti-tumor activity against TF-expressing breast cancer cells and an active ADCC compared with unmodified NK-92MI cells as controls [104].

CARN-NK cells directed against epithelial cell adhesion molecule (EpCAM), an antigen commonly identified in the surfaces of multiple cancer cells, have already demonstrated potent cytotoxicity and cytokine release (IFN-γ, perforin, granzyme B) against EpCAM-positive colorectal cancer cell lines and a xenograft mouse model [105]. In EpCAM-positive breast cancer cell lines, NK92 cells expressing EpCAM-directed CAR and IL-15 presented selective and superior cytotoxicity than unmodified NK92 cells [106]. Similarly, an HER-2-directed CD28.CD3ζCAR design expressed by NK92 cells also demonstrated anti-tumor activity against breast cancer, both in vitro and in vivo [5]. Elsewhere, HER-2-directed CAR-NK cells were found to recognize all HER-2 expressing breast cancer cell lines. The recognition included breast cancer cell lines normally resistant to NK recognition due to a lack of transmembrane ligands for NK cells or cells resistant to trastuzumab, HER-2-directed antibody, for instance, MCF-7 and MDA MB 453 that express low levels of HER-2. The intensity of the anti-tumor effect on the tested cells correlated with the HER-2 expression level [107].

Despite promising preliminary data, no clinical trial has so far been specifically dedicated to CAR-NK therapy in breast cancer patients.

### 6.3. Pancreatic Cancer

Pancreatic ductal adenocarcinoma (PDAC) is known for its abundant immunosuppressive stroma, reaching up to 70% of the tumor volume [108]. Hence, most in vivo studies of adoptive cell therapies reported unsatisfactory outcomes, with the stroma responsible for suppressing ACTs’ anti-PDAC potential. In CAR-NK cell development, multiple attempts have been made to overcome this hostile feature of PDAC.

A study of up-regulated PDAC-specific membrane markers identified folate receptor α (FRα) and death receptor 4/5 (DR4/5) as optimal targets for CAR-NK cell therapy. The study confirmed the high efficacy of FRα-directed TRAIL.CD27.CD3ζ.CAR-NK92 constructs (TRAIL, as well as DR ligands, induce apoptosis) in in vitro and in vivo xenograft mouse models of pancreatic cancer [109].

Additionally, MUC-1-directed CAR-NK cells were analyzed, in preclinical settings, in a variety of carcinomas, which led to the first clinical trial. In 2017, a phase I clinical trial examined the use of NK92-derived, lentivirally transduced, CD28.4-1BB. CAR constructs in CAR-NK cells directed against both MUC1 and PD-1. Out of eight patients with variable cancer types with positive PD-1 and MUC1 expression, e.g., lung, pancreatic, colon and ovarian cancer, seven achieved stable disease. Additionally, the study reported no serious adverse events after double targeted treatment [110]. These results were followed by a phase I/II clinical trial (NCT02839954). Patients diagnosed with GBM, hepatocellular carcinoma, pancreatic, colorectal, breast or gastric cancer, among others, who express MUC-1, were enrolled for MUC-1-directed CAR-NK cell therapy; however, as of April 2021, the results are still awaited.

Another promising target for CAR-NK therapy is ROBO-1. It has been expressed across numerous cancer types including pancreatic cancer cells [111]. ROBO-1-directed CAR-NK therapy was tested in orthotropic mouse models of pancreatic cancer. The study analyzed three groups of animals: an untreated group, a group treated with ^125^I seeds implanted into the tumor and another group receiving ^125^I brachytherapy combined with ROBO-1-directed CAR-NK cell infusions. The authors report superior tumor volume reduction in the ^125^I brachytherapy and ROBO-1-directed CAR-NK combination arm than in the ^125^I brachytherapy alone [112].

Treatment with ROBO-1-directed CAR-NK (NK92 cells, lentiviral transduction of CD8.CD3ζ.4-1BB construct, efficiency 90%) cells was described in a case report of a patient with pancreatic cancer with liver involvement. Treatment with weekly infusions and intratumoral injections (liver metastasis) of ROBO-1-directed CAR-NK cells stabilized the disease for five months. The only adverse event was the occurrence of episodes of fever following infusions [113]. Promising preclinical data prompted the launch of three phase I/II clinical trials aimed at assessing the safety and efficacy of ROBO-1-directed CAR-NK cell therapy in PDAC and other solid tumors depending on ROBO-1 expression on cancer cells, which are currently enrolling patients in China (NCT03941457, NCT03940820, NCT03931720). Interestingly, in the NCT03931720 study, an experimental arm is based on a single dose of ROBO-1-directed CAR-NK infusions with no prior conditioning therapy. Results of these trials are highly awaited.

### 6.4. Ovarian Cancer

Several studies have analyzed the effectiveness of CAR-NK cell therapy in ovarian cancer models. Mesothelin (MSLN)-directed CAR-NK92 cells demonstrated effective elimination of MSLN-expressing cell lines (OVCAR-3 and SKOV3), and of subcutaneous and intraperitoneal ovarian cancer in mice models. The therapy prolonged survival in the tested mice [114]. Elsewhere, CAR-NK cell therapy was used to target the CD24 receptor expressed on ovarian cancer cells. The anti-CD24.CAR-NK92 cells were found to be highly active against CD24-expressing ovarian cancer cell lines (SKOV3, OVCAR-3) and patient-derived cells. Finally, a dual-CAR approach with NK92 cells expressing both CD24-directed.CD28.4-1BB.CAR and MSLN-directed.CD3ζ.CAR was found to demonstrate superior effectiveness in vitro [115].

αFR-directed CAR-NK92 cells also displayed satisfactory cytotoxicity against ovarian cancer models. αFR antigen is expressed in 90% of the ovarian cancer cases. Out of three tested CAR constructs, viz. anti-αFR.CD3ζ, anti-αFR.CD28.CD3ζ and anti-αFR.CD28.4-1BB.CD3ζ, the latter presented the highest cytotoxicity, reflected in stronger degranulation and cytokine secretion, greater proliferation and longer persistence when incubated with αFR-expressing ovarian cancer cells. In the xenograft mouse models, anti-αFR.CD28.4-1BB.CD3ζ.CAR-NK92 therapy significantly prolonged survival [116].

### 6.5. Other Solid Tumors

In addition, individual studies have examined the efficacy of CAR-NK cells against other cancer types. One study found c-Met-directed CAR-NK-PBMC-derived (c-MET-directed CD8α.4-1BB.DAP12.CAR-NK-PBMC) cells to demonstrate high cytotoxicity against c-MET expressing HepG2 cells, a hepatocellular carcinoma cell line [117]. In prostate cancer, knocking out *PTPN11* (NK cell inhibitory receptor) in CAR-expressing NK-YT cells directed against PSMA (PSMA-directed.CD8α.CD28.CD3ζ.CAR-NK-YT) resulted in increased cytotoxicity against the Du-145 cell line, which is naturally resistant to NK cell killing [118].

## 7. Plausible Combination Therapies

Many therapeutics have been tested preclinically in combination with CAR-NK cells with the aim of increasing cytotoxicity and intratumoral infiltration.

The combination of cabozantinib and EGFR-directed CAR-NK92 was found to demonstrate a synergistic effect against in vitro renal cell carcinoma [119]. In addition, combination therapy with regorafenib and EpCAM-directed CAR-NK92 cells resulted in a superior anti-tumor effect against colorectal cancer than monotherapy of either treatment [109]. Inhibiting the *BRAF*(V600E) or STAT3 pathway or modulating cancer cell autophagy was also reported to sensitize cancer cells to NK cell cytotoxicity [120,121,122,123]. Interestingly, a phase II clinical trial of PD-L1-directed CAR-NK combination therapy with pembrolizumab and IL-15 superagonist (N-803), has been launched (NCT04847466) and is about to start recruiting patients in the US. The results of this trial are highly awaited.

One of the most effective drugs at inducing the expression of ligands recognized by NK cells on cancer cells is lenalidomide. It has already been tested in combination with expanded and activated unmodified NK cells in neuroblastoma and hematopoietic neoplasms studies (NCT02573896, NCT02481934, and NCT02280525) and seems to be an interesting candidate for combination with CAR-expressing NK cells. Similarly, DNA damage originating from radiation entails increased expression of NKG2D ligands on tumor cells, which activates NK cells and increases their cytotoxicity [78]. Interestingly, the widely available spironolactone was reported to upregulate the activating NKG2D ligands on colon cancer and hepatocellular carcinoma cells [124].

The TME is composed of immunocompetent cells that present negative regulation of immune responses such as MDSCs and Tregs [32,33]. Immune modulators (ipilimumab), tyrosine kinase inhibitors (sunitinib), and cytotoxic drugs (5-FU, cyclophosphamide, doxorubicin) were shown to decrease the rates of MDSCs and Tregs in the TME [125,126,127,128]. Combination therapy of MDSC and Treg-depleting agents with CAR-NK cells also seems to be a promising strategy; however, it still requires confirmation in preclinical studies.

## 8. Overview and Future Concepts

In addition to demonstrating comparable effectivity against distinct hematological malignancies as CAR-T, CAR-NK cells also appear to possess a significantly more favorable safety profile. In addition, preclinical data suggest that replacing T lymphocytes with NK cells for CAR expression may also increase treatment effectivity against a number of solid tumor types.

Studies on CAR-NK cells collectively provide a few vital indications for future development. NK-tailored CAR structure seems to be required for maximizing NK cells’ cytotoxicity. In addition, it is necessary to optimize a protocol for the expansion and activation of harvested NK cells to obtain a homogeneous suspension of clinically significant counts of memory-like, unexhausted NK cells. Furthermore, the application of CAR-NK cells in the treatment of solid tumors may require additional alterations of the NK cells beyond CAR transduction to improve trafficking and desensitize them to the immunosuppressive TME. While the results of the first CAR-NK cell clinical trials in solid tumors are still awaited, growing preclinical data indicate that this promising therapy has great potential in this setting.

## Figures and Tables

**Table 1:** Characteristics of NK cell receptors relevant in CAR-independent anti-tumor activity, mentioned in the article.

Receptor	Impact on NK Cell	Ligands	Function
CD16	Activating	Fc domain of IgG	ADCC initiation through the classical ITAM pathway. Its activation is sufficient to ignite cytotoxicity and cytokine production in resting NK cells, regardless of the adaptor particle.
DNAM-1	Activating	CD112, CD155	Expressed on NK cells, T cells and monocytes. Its signaling promotes cellular adhesion.
KIR	Activating/Inhibitory	HLA class I	Its impact on NK cells depends on the isoform. Inhibitory isoforms outweigh activating ones in the affinity to HLA class I ligands.
NKG2A	Inhibitory	HLA-E	Forms complexes with CD94 and through the ITIM motif transduces inhibitory signals. Has a higher affinity to HLA-E ligands than the CD94/NKG2C complex.
NKG2C	Activating	HLA-E	Forms complexes with CD94 and associates with DAP12 for an activating signal transduction. Has a lower affinity to HLA-E ligands than the CD94/NKG2A complex.
NKG2D	Activating	MICA, MICB ULBP-1-6	Expressed on the surface of NK, γδ T and CD8+ T cells. Associates with the DAP10 homodimer. Binds ligands upregulated by infected, stressed or transformed cells.
NKp30	Activating	HA/HS/GAGs	Member of the NCR family. Expressed constitutively on mature, resting or activated NK cells and some populations of T cells. Downregulated on memory-like NK cells.
NKp44	Activating	HA/HS/GAGs	Found only on activated NK cells. Associates with the DA12 homodimer for cytokine release ignition.
NKp46	Activating	HA/HS/GAGs	Uniquely expressed on NK cells. Member of the NCR family. Triggers cytotoxic reaction.
PD-1	Inhibitory	PDL-1, PDL-2	Expressed on various immune cells. Binds to ligands present across multiple cancer types leading to NK cell exhaustion and cancer immune escape. The most abundantly expressed by mature NK cells.
TIGIT	Inhibitory	CD112, CD113, CD155	Expressed in NK and T cells. Among others, binds to the CD155 ligand rarely expressed on healthy cells, but found abundantly in cancer cell lines and human neoplasms. TIGIT blockade prevents NK and T cell exhaustion and tumor cell escape.
2B4	Activating	CD48	Expressed on diverse immune cells beyond NK cells. Binds to CD48, widely expressed on cells of hematopoietic origin. In immature NK cells, can act as an inhibitor.

**Table 2:** Co-receptors used the most often in CAR design in CAR-NK cell studies.

Co-Receptor	Function in a CAR Particle	Description
CD28	Co-stimulation	Essential for T cell activation. Improves anti-tumor activity and persistence at a low effector to target ratio. Induces IL-2 production and CD4^+^ T cell expansion. In CAR-T cells, induces central and effector memory phenotypes. Responsible for recruitment of Tregs limiting CAR-T activity. Not expressed in unmodified NK cells. Used as co-stimulatory receptor with CD3ζ in CAR-NK cells, gives superior results than CD3ζ.CAR alone.
CD3ζ	Signaling	Expressed in unmodified NK and T cells. Used in both CAR-T and CAR-NK cells. Contains three ITAM motifs for signal transduction. Initiates cell killing. In dimers, transmits CD16 signal for ADCC initiation. Stimulates proliferation.
DAP10	Co-stimulation	An adaptor molecule. Does not have an ITAM motif, unable to transduce the signal. Expressed in unmodified T and NK cells. Interacts with activating NKG2D receptor. Involved in NK cells activation.
DAP12	Co-stimulation Signaling	An adaptor molecule. Has one ITAM motif. Superior signaling potential to CD3ζ. Interacts with NKG2C, NKp44, KIR receptors. Not expressed in unmodified T cells. Involved in NK cells activation.
2B4	Co-stimulation	Expressed in unmodified NK cells. In CAR-NK cells, induces cytokine production and invasiveness.
4-1BB	Co-stimulation	Essential for T cell proliferation and survival. Enhances the proliferation of CD8+ and CD4+ T cells. Induces IL-2 production. Prolongs T cell division. Prolongs CAR-T cell persistence. Not expressed in unmodified NK cells. Used as co-stimulatory receptor with CD3ζ in CAR-NK cells. Gives superior results than CD3ζ.CAR alone.

## Data Availability

Not applicable.
